# PFO-DBT:MEH-PPV:PC_71_BM Ternary Blend Assisted Platform as a Photodetector

**DOI:** 10.3390/s150100965

**Published:** 2015-01-07

**Authors:** Qayyum Zafar, Zubair Ahmad, Khaulah Sulaiman

**Affiliations:** Low Dimensional Materials Research Centre (LDMRC), Department of Physics, Faculty of Science, University of Malaya, 50603 Kuala Lumpur, Malaysia; E-Mails: qayyumzafar@siswa.um.edu.my (Q.Z.); khaulah@um.edu.my (K.S.)

**Keywords:** PFO-DBT, MEH-PPV, ternary blend, organic photodetector

## Abstract

We present a ternary blend-based bulk heterojunction ITO/PEDOT:PSS/PFO-DBT:MEH-PPV:PC_71_BM/LiF/Al photodetector. Enhanced optical absorption range of the active film has been achieved by blending two donor components *viz.* poly[2,7-(9,9-di-octyl-fluorene)-alt-4,7-bis(thiophen-2-yl)benzo-2,1,3-thiadiazole] (PFO-DBT) and poly(2-methoxy-5(2′-ethylhexyloxy) phenylenevinylene (MEH-PPV) along with an acceptor component, *i.e.*, (6,6)-phenyl-C_71_ hexnoic acid methyl ester. The dependency of the generation rate of free charge carriers in the organic photodetector (OPD) on varied incident optical power density was investigated as a function of different reverse biasing voltages. The photocurrent showed significant enhancement as the intensity of light impinging on active area of OPD is increased. The ratio of I_light_ to I_dark_ of fabricated device at −3 V was ∼3.5 × 10^4^. The dynamic behaviour of the OPD under on/off switching irradiation revealed that sensor exhibits quick response and recovery time of <800 ms and 500 ms, respectively. Besides reliability and repeatability in the photoresponse characteristics, the cost-effective and eco-friendly fabrication is the added benefit of the fabricated OPD.

## 1. Introduction

Solution-processable organic photodetectors have attracted significant R&D efforts as a promising alternative to inorganic semiconductor devices [1,2] by virtue of their low-cost, device architectural flexibility and large-scale fabrication capability [3–5]. To date, the organic bulk heterojunction approach, particularly intimately blended D/A binary blend is the dominant theme for image sensing application [6,7]. The operating principle of BHJ OPDs relies on the efficient dissociation of the photoinduced geminate carrier pairs at D/A intermolecular contacts. Photodetection parameters intensively rely on electrical properties including HOMO/LUMO energy levels, the charge carrier mobility of the donor (D) and acceptor (A) materials as well as the structure of the photodiode. Hoppe *et al.* report that enhanced interfacial area between D/A phases within BHJ device design enables efficient charge separation as compared to planar interface of bilayer devices [8]. For bulk heterojunction approach, the photons absorbed throughout the interpenetrating network of D/A materials contribute to the photogenerated current. Composite of judiciously chosen pair of conjugated polymer donors and acceptor material thus yield high photoinduced charge generation and pronounced charge transfer [9].

Several binary blend-based photo sensing platforms have been proposed so far. In our previous studies, we have explored a variety of D/A combinations for the applications in organic solar cells and OPDs [10–13]. The main challenge of OPDs design engineering is to develop the sensing layer that can harvest a wide range of incident photon energies. Optoelectronic devices based on BHJ consisting of two components, exhibit limited light-harvesting range. Usually, the acceptor materials in D/A binary blend contribute very little to the light harvesting, furthermore, their selection range to make a suitable D/A combination is also limited [14]. Light is mainly absorbed by donor moiety (*i.e.*, conjugated polymer) in the blend only [15]. Hence in principle, an ideal donor polymer should exhibit high hole mobility coupled with the broad absorption spectra to match with visible solar terrestrial radiation [16]. Ternary blend bulk heterojunction (BHJ) provides a distinct platform and an alternate route to enhance the absorption bandwidth of OPD'S while maintaining the ease and benefits of a single photoactive layer. Summation of the individual absorption spectra of two distinct donors leads to a noticeably strong and wide absorption spectrum spanning over the long range. Yet another benefit of incorporating a second donor material within the photoactive layer is that charge transfer barriers are believed to reduce in the resulting D/A ternary blend as well [17]. Ternary blend approach has already been utilized to enhance the power conversion efficiency (PCE) of solar cells [18–20], however much less attention has been paid to utilize it for photodetection application.

In our previous studies, we demonstrated MEH-PPV:VOPCPhO [11] and MEH-PPV:Alq_3_ [12] binary blend-based photodetectors. MEH-PPV exhibits fairly good hole mobility and environmental stability [21]. However, the photosensitivities and the photo to the dark current ratios of both binary blends-based OPDs were relatively lower. In the present work, we aim to demonstrate more sensitive visible wavelength photodetector with two donor components *i.e.*, MEH-PPV and PFO-DBT in order to enhance the photosensitivity and the photo to the dark current ratio. Conjugated conducting polymer PFO-DBT is used as an electron donor component in photoactive layer of organic solar cells. However, it is ineffective to harvest light in shorter wavelength ranges near 450 nm, thereby limiting the overall device performance. MEH-PPV on the other hand harvests light efficiently in this visible wavelength range. Fullerene derivative PCBM, undoubtedly remains an ubiquitously utilized acceptor material by virtue of its excellent ability to induce ultrafast electron transfer and transport properties, in contrast to VOPCPhO and Alq_3_. In the present study, PC_71_BM has been utilized by virtue of its enhanced visible light absorption as compared to PC_61_BM [22]. The motivation to choose these MEH-PPV, PFO-DBT and PC_71_BM materials, is continuation and improvement in our previous studies. Ternary blend seems to be a potential approach for tailoring the sensing parameters of OPD which is otherwise not possible by using binary BHJ approach. In our present systematic study, we therefore aim to achieve relative performance increase by increasing the spectral sensitivity of the photoactive film, which is prerequisite for improvement in sensing parameters of the OPD. The spectral responses of the PFO-DBT:PCBM and MEH-PPV:PCBM devices have previously been investigated by Zhou *et al.* [23]. It is found that the photoresponse of PFO-DBT:PCBM device is 50–60 nm more toward the higher wavelength as compared to the MEH-PPV:PCBM device. This gives the clue that if we make the blend of PFO-DBT:MEH-PPV:PCBM then the improved spectral response towards the higher wavelengths can be obtained. A ternary blend of PFO-DBT:MEH-PPV:PC_71_BM is therefore expected to yield improved sensing parameters, *i.e.*, better photoresponsivity and an enhancement in photo to dark current ratio.

## 2. Experimental

**Figure 1.**
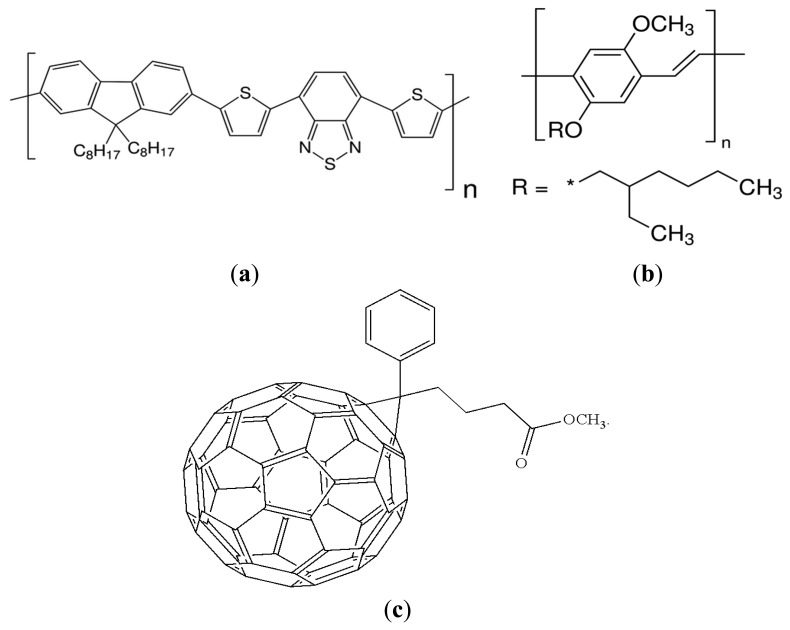
The molecular structure of (**a**) PFO-DBT (**b**) MEH-PPV and (**c**) PC_71_BM.

Poly[2,7-(9,9-di-octyl-fluorene)-alt-4,7-bis(thiophen-2-yl)benzo-2,1,3-thiadiazole] (PFO-DBT) and [6,6]-phenyl-C_71_-butyric-acid methyl ester (PC_71_BM) were obtained from a commercial supplier Luminescence Technology Corp. (Taiwan, China). No further purification was done and they were used as received. Poly[2-methoxy-5-(2′-ethylhexyloxy)-*p*-phenylenevinylene] (MEH-PPV) was synthesized by established Gilch's polymerization reaction [24]. The aqueous solution of poly(3,4-ethylenedioxythiophene):poly(styrene sulfonate) (PEDOT:PSS) with PH ∼1000 and conductivity 900–1000 S/cm was commercially obtained from H.C. Starck (Goslar, Germany). Molecular structures of PFO-DBT, MEH-PPV and PC_71_BM are depicted in Figure 1. Commercially available ITO substrates (25 mm × 25 mm slides, sheet resistance ∼12 Ω/sq), were cleaned according to a well-established protocol *i.e.*, by ultrasonic agitation in acetone, ethanol, isopropyl alcohol and DI water, respectively, and later substrates were dried clean by blown nitrogen. An anode buffer layer of PEDOT:PSS was then spun-coated onto ITO substrates at 3000 rpm for 30 s. The resulting transparent (∼40 nm thick) PEDOT:PSS thin film was annealed at 120 °C for half an hour. Thirty mg/mL concentrated solutions for PFO-DBT, MEH-PPV and PC_71_BM each, were prepared separately in chloroform by stirring them overnight at room temperature. The photoactive layer of PFO-DBT:MEH-PPV:PC_71_BM ternary blend with optimum ratio was spun cast on top of PEDOT:PSS layer. Spin speed was maintained at 4000 rpm, yielding a thickness of 120 nm. Photoactive layer deposition was followed by baking at 120 °C for half an hour. The optimum mixing ratio (by volume) of donor binary blend was selected by the help of photoluminescence (PL) study. A thin film of LiF (∼10 Å) was used between active layer and top electrode to enhance the performance of the OPD. Two of the several suggested advantages of LiF coating are: (a) lowering of the effective work function of top Al cathode and (b) protection of photoactive layer from hot Al atoms during its deposition [25]. Both LiF and aluminium (Al) were thermally deposited via shadow mask. The fabricated devices were in circular shapes with diameter ∼1 mm. The evaporation rates for LiF and Al cathodes were controlled to be 0.1 and 3 nm/s to achieve a thickness of 0.8 and 100 nm, respectively. Finally the fabricated devices were annealed at 120 °C for 30 min. The entire fabrication process of OPD was carried out into a glove-box with inert N_2_ atmosphere, except for the PEDOT:PSS aqueous solution coating. The optical absorption study in visible spectral range was carried out using a UV-Vis-NIR spectrophotometer (Shimadzu UV-3101PC). The photoluminescence (PL) dynamics of active film in the visible and near-infrared-spectral ranges were studied by using RENISHAW in via a Raman microscope instrument. The RENISHAW in via Raman microscope instrument uses the 325 nm laser wavelength and the detector limits of this system are 400–1000 nm. The I-V characteristics of the OPD were measured under different light intensities (0–150 mW/cm^2^) using an Oriel 67,005 solar simulator and were recorded by a Keithley 236 source measuring unit (SMU).

## 3. Results and Discussion

**Figure 2.**
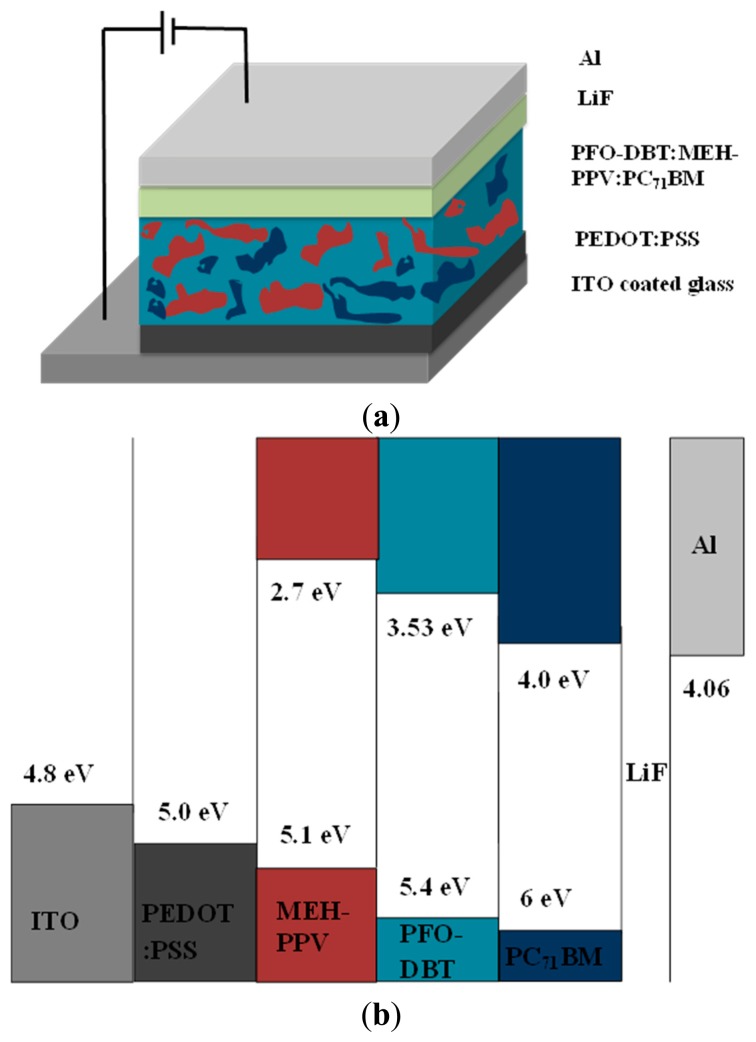
(**a**) Schematic view of the device geometry, ITO/PEDOT:PSS/PFO-DBT:MEH-PPV:PC_71_BM/LiF/Al with ternary blended donor-acceptor structure and (**b**) the energy bands of their component materials.

The layer stack of the fabricated OPD (ITO/PEDOT:PSS/PFO-DBT:MEH-PPV:PC_71_BM/LiF/Al) and energy level diagram of the components have been depicted in Figure 2a,b, respectively. Figure 2b shows the energy levels of PFO-DBT, MEH-PPV and PC_71_BM and work functions of ITO and Al. The HOMO/LUMO energy levels were obtained from material safety data sheets (MSDS) provided by the manufacturer of the materials. Same values have also been reported by other researchers in the literature [23,26,27]. Inorganic fluorides such as LiF have good electron extraction ability from active layer, further they serve as a hole blocking layer as well [28]. PEDOT:PSS on the contrary, serves as a buffer layer for hole collection at the ITO anode. The energetic position of the frontier levels of the D/A materials used in the present work ensures better flow of photogenerated charges to the appropriate direction. The potential offset at the D/A interfaces provide adequate energy to dissociate electron-hole pairs. The transport and collection of mobile charge carriers at the electrodes is then facilitated by the external bias and built-in electric field between PEDOT:PSS and Al work functions.

**Figure 3.**
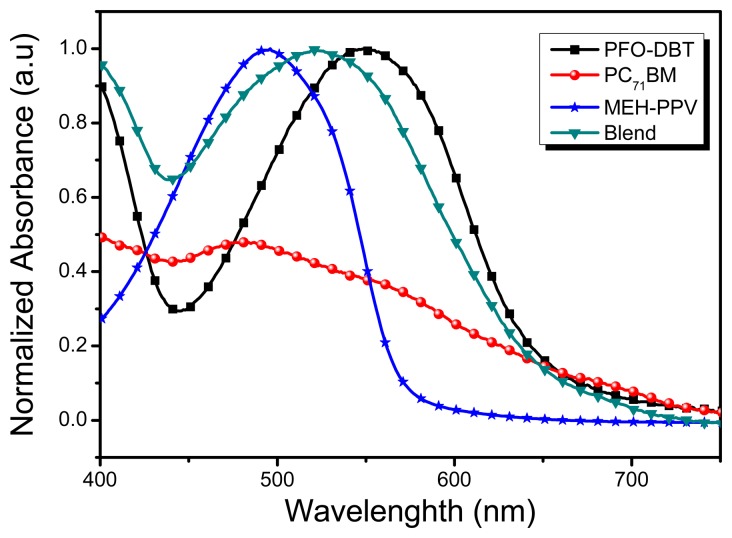
UV-Vis spectra of PFO-DBT, MEH-PPV, PC_71_BM neat materials and their ternary blend thin film.

Figure 3, compares the optical absorption spectra of the photoactive donor polymers: MEH-PPV, PFO-DBT and acceptor moiety PC_71_BM. The peak optical absorption of MEH-PPV is at 510 nm, which indicates that substantial portion of incident visible light is not harvested by MEH-PPV alone. The motivation here is to extend the absorption profile of the photoactive film of OPD still further toward longer wavelengths. The approach used to achieve a comprehensive coverage of the solar spectrum is the addition of other polymeric donor material with a complimentary absorption profile. For instance, PFO-DBT exhibits a light complementary absorption to that of MEH-PPV. The UV-VIS spectroscopic data confirms that PFO-DBT dominates the spectral absorbance above 590 nm, where either of MEH-PPV and PC_71_BM fails to absorb photons. The absorption spectrum of PFO-DBT exhibits two maxima; the first is located at 384 and the second at 540 nm. Yong *et al.* [29] suggest that absorption band at smaller wavelengths is attributed to fluorine segment and the additional longer wavelength absorption band is attributed to DBT unit of the PFO-DBT polymer. By virtue of complimentary absorption profile, the inlay of PFO-DBT to the MEH-PPV-PC_71_BM is believed to yield a broader and consistent absorption spectrum. Further, PC_71_BM exhibits strong absorption ability at shorter wavelengths (350–500 nm) [30], hence it is potentially adopted as an acceptor material in BHJ solar cells with relatively higher power conversion efficiency (PCE) [31–33]. The spectral response of the MEH-PPV:PCBM and PFO-DBT:PCBM based photovoltaic devices have already been reported [23]. Further, it can be cross-referenced that the spectral response is in good agreement with the independent material's absorption spectra.

**Figure 4.**
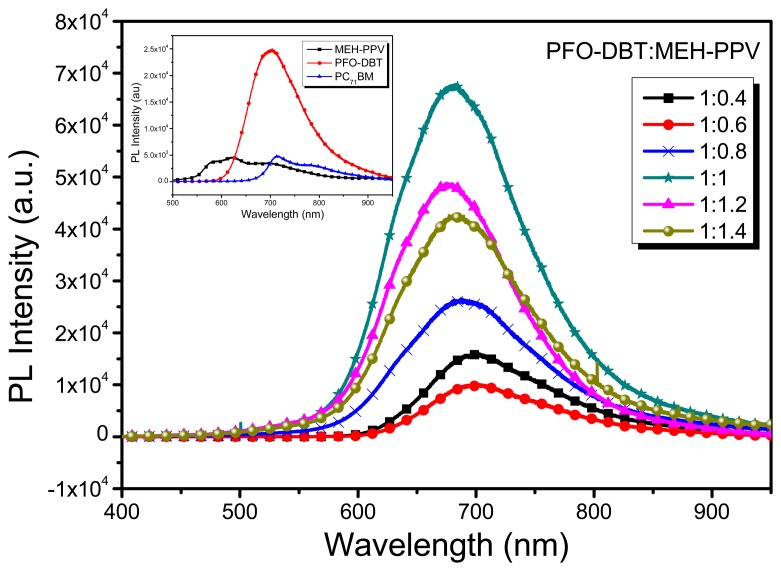
PL spectra of PFO-DBT: MEH-PPV blends at different volumetric ratio. (Inset) PFO-DBT, MEH-PPV and PC_71_BM PL spectra, when studied independently.

The photoluminescence measurement (PL) of the thin films of PFO-DBT:MEH-PPV binary blend was obtained by an excitation wavelength of 325 nm in the range from 400 to 1000 mm. Figure 4 (inset) depicts the PL spectra of thin films of MEH-PPV, PFO-DBT and PC_71_BM independently. Figure 4, presents the PL spectrum of binary blend for six different volume ratios (1:0.4, 1:0.6, 1:0.8, 1:1, 1:1.2 and 1:1.4). The photoluminescence (PL) of binary blend has been studied in order to obtain optimized volumetric ratio providing enhanced photoinduced exciton splitting efficiency within the photoactive layer. The binary blend exhibits strong emission peak at 700 nm wavelength for all volumetric ratios. At 1:0.6 volumetric ratio of the binary blend, the PL intensity is markedly quenched, indicating a more efficient charge transfer between the organic materials. Emission occurs when the photogenerated excitons recombine emissively instead of splitting into free carriers. Quenching in emission spectra on contrary is an indicator of how well excitons split into free charges instead of undergoing emissive recombination [8]. Subsequently improvement in photocurrent can thus be realized for 1:0.6 optimized volumetric ratio of binary blend. PC_71_BM was incorporated as an acceptor material in the binary donor blend. 1:0.8 volumetric fraction of D:A has been used in the ternary blend. As it is well known, the absorption of incident photon flux in donor moiety of BHJ blend generates columbically bound electron-hole bound pairs called excitons. These photogenerated excitons can either relax back to the ground state or dissociate at D/A interface into free electrons and holes. Since the photoresponse of OPD results from photogeneration of free charge carriers so exciton must be dissociated to mobile charges. Splitting of photogenerated excitons is caused by the difference in electron affinities at the D/A interface of BHJ blend. Band diagram of the ternary blend (depicted in Figure 2b) indicates desirable offsets in frontier orbital energies (HOMO/LUMO levels) that are sufficient to overcome the columbic forces present between the photoinduced excitons. The same built-in potential is the reason of the electron drift, towards LUMO of the material with the larger electron affinity (PC_71_BM acceptor phase) and hole drift, toward HOMO of the materials with the lower ionization potential (MEH-PPV and PFO-DBT donor phases). These separated charges are then transported to the electrodes to create photocurrent in external circuit. The whole photodetection phenomenon can thus be summed up as the generation, separation and transport of charge carriers towards their respective electrodes.

**Figure 5.**
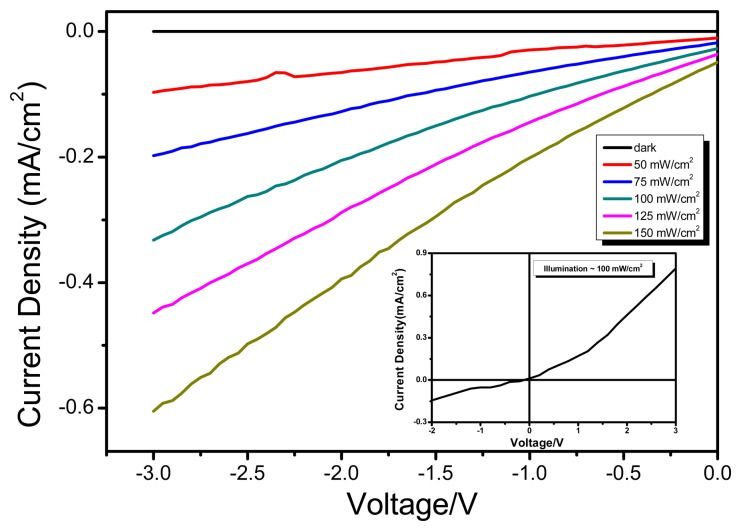
The photoresponce characteristics of the OPD (ITO/PEDOT:PSS/PFO-DBT:MEH-PPV:PC_71_BM/LiF/Al) illustrating photocurrent dependence on light illumination levels. The measurements were recorded under different light intensities from 0 to 150 mW/cm^2^, at room temperature. Inset shows the I-V characteristics of the OPD under forward and reverse biased condition at room temperature.

**Figure 6.**
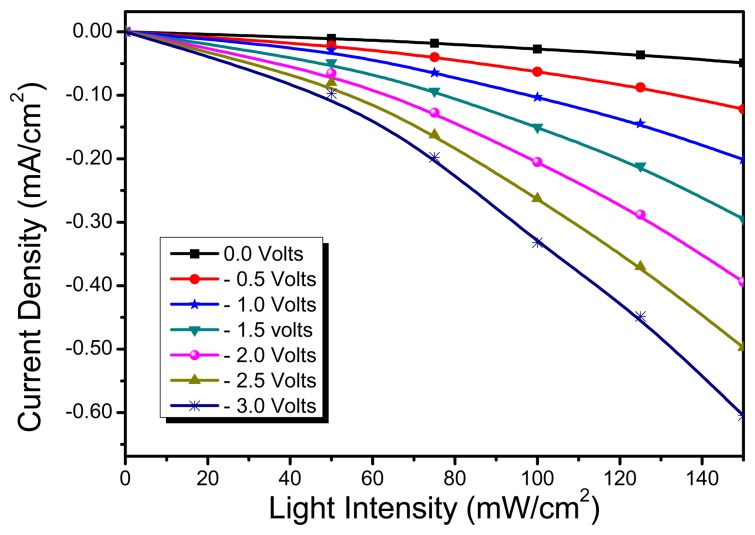
Light intensity v's. current density measurements for ITO/PEDOT:PSS/PFO-DBT:MEH-PPV:PC_71_BM/LiF/Al photodetector at different bias voltages (0 to −3 V) at room temperature.

Typically, the photodetector functions as a photodiode in photoconductive mode and is usually operated in reverse biased configuration. The use of two electrodes (ITO and Al) with different work function, ensures the diodic behaviour as is evident from characteristic IV curves of diode Figure 5 (inset). With the increase in optical power density, the generation rate of mobile charge carriers increases and resultantly photodetector outputs an increased photocurrent. This aforementioned phenomenon forms the basis of operation principle of photo detector. To evaluate the performance of the ternary blend based OPD, its reversed biased current-voltage (I-V) characteristics were investigated. Figure 5, shows the photoresponse of the OPD when exposed to simulated light of varied illumination levels. It can be observed that the reverse biased current in dark condition (I_dark_) is quite low as compared to the photo induced current (I_light_) under the influence of simulated light illumination. It is also evident from Figure 5 that with higher influx of incident photons on OPD; a higher density of separated charges approaches the electrodes, giving rise to pronounced magnitude of photocurrent. Furthermore, from Figure 6, it can be inferred that magnitude of photoresponse is increased with the increase in the electric field (sourced by external reverse bias voltage). In fact, the couple of bottlenecks to better photoresponse are: (1) enhanced photon absorption; (2) efficient creation and splitting of excitons into independent holes and electrons; (3) efficient transportation of charges to collection electrodes. The bulk heterojunction blend approach helps only to improve both photon absorption and exciton dissociation. However the migration of separated charges towards electrodes is external field assisted. External biasing serves as a driving force for transportation of charge carriers through interpenetrating D/A networks. The transportation becomes faster and efficient at higher order of potential bias, resulting in improved photoresponse. However at lower biasing, mobile charges are more likely to get trapped or encounter recombination rather than being collected at the electrodes.

Table 1, compares the sensing parameters of the present ternary blend based OPD and binary blend based OPDs previously reported by our research group. Sensing parameters have been compared at −3 V applied bias. The ratio of I_light_ to I_dark_ of fabricated device at −3 V is ∼3.5 × 10^4^, which is significantly higher as compared to our previous study [11]. Similarly, the response/recovery times of the sensor have also showed marked improvement comparatively. The photoresponsive parametrs of ternary blend based OPD have also been compared with those of MEH-PPV:PCBM binary blend based OPD, reported earlier by Barai [34]. Table 2, signifies that with the incorporation of PFO-DBT in MEH-PPV:PCBM blend, the fabricated OPD has shown marked improvement in its photo response characteristics. For reference, typically the response times and responsivity for bulk heterojunction based OPDs are in the order of ms and mA/W respectively [35–37].

**Figure 7.**
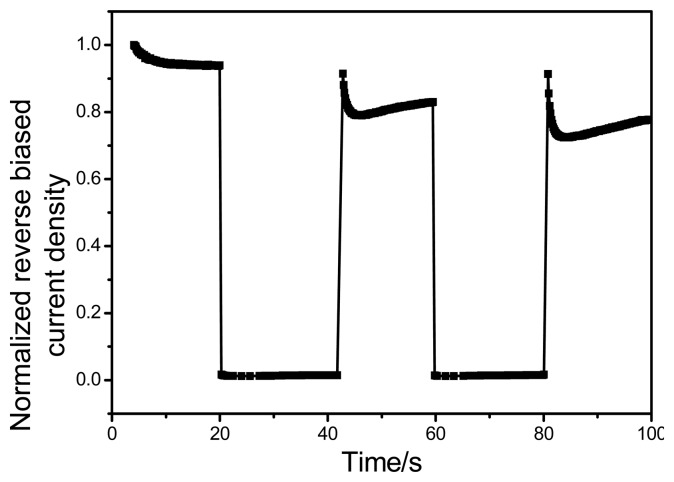
The response time of the ITO/PEDOT:PSS/PFO-DBT:MEH-PPV:PC_71_BM/LiF/Al under pulsed optical illumination with the intensity of 100 mW/cm^2^ at −3 V biasing. The maximum value of current density was ∼0.4 mA/cm^2^ under 100 mW/cm^2^ at −3 V biasing.

The temporal response is another important parameter of the photodetector and is determined by the average exciton lifetime in photoactive film. Reasonably fast response times can be achieved by incorporating thinner photoactive films in OPDs [38]. The fabricated OPD demonstrates a sharp change in its magnitude of photocurrent in response to periodic pulsed simulated solar light (irradiance ∼100 mW/cm^2^, pulse width equals 20 s) as shown in Figure 7. The presented sensor has been investigated as a whole visible light detector, therefore a solar simulator has been used to impinge whole visible spectrum on the sensing area instead of a monochromator. The photodetector was externally biased at −3 V, while probing the time dependent photoresponse characteristics as a function of light modulation ON/OFF. As explained in the photocurrent generation mechanism earlier, when light impinges on the active film it causes a progressive rise of the photocurrent amplitude by virtue of photogenerated charges. The resulting time-resolved photocurrent response is gradual, stable and repeatable as evidenced by three successive cycles of abrupt switching between ON and OFF states of illumination. The initial photocurrent spike observed during light exposure is due to the momentary surge in illumination level, which is commonly observed when a halogen bulb is switched on. As the illumination level of the bulb stabilizes to 100 mW/cm^2^, photocurrent also tends to stabilize after the momentary initial spike. For our present OPD, in response to sudden light irradiation, the photocurrent density progressively increased from ∼−0.168 nA/cm^2^ to −0.330 mA/cm^2^. The normalized transient reverse biased photocurrent behavior has been depicted in Figure 7. The time required for the photocurrent to increase from 10% to 90% of the final settled value or *vice versa* is generally defined as the rise and decay time (t_r_ and t_d_), respectively [39]. Figure 7, reveals the operation speed of OPD, in present case the rise time (t_r_) is about 800 ms, and fall time (t_f_) is 500 ms. For reference, typically the response times for bulk heterojunction based OPDs are in the order of (10^−8^ to 10^−3^) s [40]. Similar fast response time are observed in the case of inorganic photodiodes, Liu *et al.* for instance, reported response time on the order of microseconds for photodiode based on high quality ZnO epitaxial films on sapphire substrates [41]. It is worth noting that the ternary blend based photodetector exhibited fast response and recovery times as compared to binary blend based devices previously reported by our group (refer to Table 1). The repeatability and reproducibility of the response/recovery time measurements were investigated for five devices and similar robust stability and reproducibility in response has been observed.

**Figure 8.**
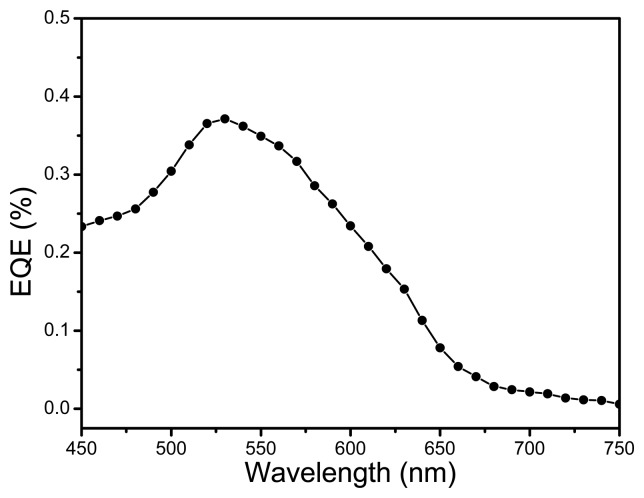
EQE spectrum of the ITO/PEDOT:PSS/PFO-DBT:MEH-PPV:PC_71_BM/LiF/Al photodetector.

Figure 8 depicts the external quantum efficiency (EQE) spectrum for the fabricated OPD. The EQE spectrum was obtained by using a xenon lamp (150 W Oriel), a monochrometer and with the help of a calibrated Si-photodetector. It can be inferred from the spectrum that the ternary blend helps to enhance the light utilization efficiency of the active film, which is vital for improved sensing parameters. EQE spectrum matches well with the visible absorption spectrum. Compared to the absorption spectrum of the ternary blend, the photocurrent from 450 nm to 650 nm is mainly due to the two donor materials *i.e.*, MEH-PPV and PFO-DBT. Light harvesting at shorter wavelength *i.e.*, near 450 nm is mainly contributed by MEH-PPV phase whereas at higher wavelengths, PFO-DBT phase contributes more towards light harvesting. The photosensitivity spectra of the binary blend based photodetectors of MEH-PPV-PCBM and PFO-DBT-PCBM have already been investigated by Zhou *et al.* [23]. It can be cross-referenced that the EQE spectrum of the ternary blend covers the detection range from 450 nm to 650 nm which consist of the sensitivity range of the both photodetectors *i.e.*, MEH-PPV-PCBM and PFO-DBT-PCBM.

## 4. Conclusions

A solution processable ternary blend-based OPD with improved sensing performance has been successfully demonstrated without sacrificing the attractive simplicity of single photoactive layer fabrication design. Marked improvement in sensing parameters, *i.e.*, *I*_Ph_/*I*_Dark_ and switching time between stable dark and illuminated states have been observed. The reason for the higher sensitivity may be attributed to judicious component and compositional selection of the ternary blend. The addition of PFO-DBT in the MEH-PPV:PC_71_BM blend significantly enhances the absorption profile of photoactive layer, thereby increasing the magnitude of photocurrent and improvement in sensing parameters of OPD. Time resolved measurements of photocurrent response as a function of pulsed illumination revealed response and recovery times as fast as 800 ms and 500 ms, respectively.

## Figures and Tables

**Table 1.** Comparison of the sensor's parameters at −3 V biasing at 100 mW/cm^2^.

**OPDs**	***I*_Ph_/*I*_Dark_**	**Responsivity**	**Response and Recovery Time**
**MEH-PPV:VOPCPhO [11]**	5.9	5 × 10^−4^ mA/W	∼4 s both
**PFO-DBT:MEH-PPV:PC_71_BM**	3.5 × 10^4^	3.9 mA/W	∼800 and ∼500 ms

**Table 2.** Comparison of the binary and ternary blend based sensor's parameters at reverse biasing.

**Current Density (A/cm^2^**)	**MEH-PPV:PCBM [34] Bias = −3.5 V**	**PFO-DBT:MEH-PPV:PC_71_BM Bias = −3.0 V**
**Dark Current Density (D)**	−2.49 E−7	−1.7 E−8
**Photo Current Density (P)**	−3.79 E−5	−6.0 E−4
**Ratio = P/D**	152.29	3.5 E4
